# Spike-Timing-Dependent Plasticity and Short-Term Plasticity Jointly Control the Excitation of Hebbian Plasticity without Weight Constraints in Neural Networks

**DOI:** 10.1155/2012/968272

**Published:** 2012-12-30

**Authors:** Subha Fernando, Koichi Yamada

**Affiliations:** ^1^Information Science and Control Engineering, Graduate School of Engineering, Nagaoka University of Technology, 1603-1 Kamitomioka-machi, Nagaoka, Niigata 940-2188, Japan; ^2^Management and Information Systems Science, Faculty of Engineering, Nagaoka University of Technology, 1603-1 Kamitomioka-machi, Nagaoka, Niigata 940-2188, Japan

## Abstract

Hebbian plasticity precisely describes how synapses increase their synaptic strengths according to the correlated activities between two neurons; however, it fails to explain how these activities dilute the strength of the same synapses. Recent literature has proposed spike-timing-dependent plasticity and short-term plasticity on multiple dynamic stochastic synapses that can control synaptic excitation and remove many user-defined constraints. Under this hypothesis, a network model was implemented giving more computational power to receptors, and the behavior at a synapse was defined by the collective dynamic activities of stochastic receptors. An experiment was conducted to analyze can spike-timing-dependent plasticity interplay with short-term plasticity to balance the excitation of the Hebbian neurons without weight constraints? If so what underline mechanisms help neurons to maintain such excitation in computational environment? According to our results both plasticity mechanisms work together to balance the excitation of the neural network as our neurons stabilized its weights for Poisson inputs with mean firing rates from 10 Hz to 40 Hz. The behavior generated by the two neurons was similar to the behavior discussed under synaptic redistribution, so that synaptic weights were stabilized while there was a continuous increase of presynaptic probability of release and higher turnover rate of postsynaptic receptors.

## 1. Introduction

Even though Hebbian synaptic plasticity is a powerful concept which explains how the correlated activity between presynaptic and postsynaptic neurons increases the synaptic strength, its value has been diminished as a learning postulate because it does not provide enough explanation how synaptic weakening occurs. In simple mathematical interpretation of Hebbian learning algorithm, an increase of the synaptic strength between two neurons can be seen if their activity is correlated otherwise it is decreased [1]. This interpretation to Hebbian plasticity allows boundless growth or weakening of synaptic strength between the two neurons [2]. Even though Hebbian plasticity has been supported by the biological experiments on long-term plasticity, it is still not completely understood how Hebbian plasticity can avoid synaptic saturation and bring the competition between synapses to balance the excitation of Hebbian neurons. Normalization of weight [2], BCM theory [3], and spike timing-dependent plasticity (STDP) [4] are the most biologically significant mathematical mechanisms that have been discussed in the literature to address this issue effectively. Weight normalization has been introduced either in additive or multiplicative modes to scale the synaptic weights and to control the continuous growth or weakening of synaptic strength; however, these user-defined weight constraints significantly affect the dynamic behavior of the applied neural network and limit the performance of learning [5]. BCM theory is another significant approach that explains synaptic activity as a temporal competition between input patterns. Synaptic inputs that drive postsynaptic firing to higher rate than a threshold value result in an increase of synaptic strength while inputs that make postsynaptic firing to lower rate than the threshold value result in a decrease of synaptic strength. BCM approach has mainly considered instantaneous postsynaptic firing frequencies for its threshold updating mechanism instead of spike arrival time to the synapses. As per the recent literature, it has recognized STDP [4] as a key mechanism of how information is processing in the brain. STDP is a form of long-term plasticity that merely depends on the relative timing of presynaptic and postsynaptic action potentials [6, 7]. Although the process and the role of STDP in information passing in some area of the human brain in the development stages are still not clear [8, 9], it has been shown that average case versions of the perception convergence theorem hold for STDP in simple models of spike neurons for both uncorrelated and correlated Poisson input spike trains. And further it has shown that not only STDP changes the weight of synapses but also STDP modulates the initial release probability of dynamic synapses [10]. Moreover, STDP has been tested on a variety of computational environments, especially to balance the excitation of Hebbian neurons by introducing synaptic competition [11–13] and to identify the repetitive patterns in a continuous spike trains [14, 15]. These experimental studies on synaptic competition using STDP are conducted in two forms: additive form and multiplicative form. In additive form, for example, as in [11], synapses competed against each other to control the timing of postsynaptic firing but this approach assumed that synaptic strength does not scale synaptic efficacy and hard constraints were used to define the efficacy boundaries. In the multiplicative form synaptic scaling was separately introduced to synaptic weight as a function of postsynaptic activity [12, 13]. However, because of the reduced competition between synapses, for strong spike input correlations all synapses stabilized into similar equilibrium. In sum, many applications based on STDP to control the excitation of Hebbian neuron depend on the user-defined constraints on weight algorithm which ultimately limit the performance of learning. To alleviate this limitation in the learning process of using hard weight constraints, another significant approach has been discussed in the literature to remove the correlation in input spike trains by using recurrent neural networks [16]. Their results claim the possibility of reducing the correlation in the spike inputs by recurrent network dynamics. The experiment was conducted on two types of recurrent neural networks; with purely inhibitory neurons and mixed inhibitory-excitatory neurons. At low firing frequencies, response fluctuations were reduced in recurrent neural network with inhibitory neurons when compared to feed-forward network with inhibitory neurons. Moreover, in the case of homogeneous excitatory and inhibitory subpopulation, negative feedback helps to suppress the population rate in both recurrent neural network and feed-forward network. Because inhibitory feedback effectively suppresses pairwise correlations and population rate fluctuations in recurrent neural networks, they suggested using inhibitory neurons to de correlate the input spike correlations. Moving one step further by combining the underlying concepts in [17, 18], that is, using nonlinear temporally asymmetric Hebbian plasticity and recent experimental observation of STDP in inhibitory synapses, Luz and Shamir [19] have discussed the stability of Hebbian plasticity in feed-forward networks. Their findings supported the fact that temporally asymmetric Hebbian STDP of inhibitory synapses is responsible for the balance the transient feed-forward excitation and inhibition. Using STDP rules, the stochastic weights on inhibitory synapses were defined to generate the negative feedback and stabilized into a unimodal weight distribution. The approach was tested on two forms of network structure; feed-forward inhibitory synaptic population and feed forward inhibitory and excitatory synaptic population. The former structure converged to a uniform solution for correlation input spikes but later destabilized and excitatory synaptic weights were segregated according to the correlation structure in input spike train. Even though the proposed model in the presence of inhibitory neurons of the learning is more sensitive to the correlation structure, the stability of the network is needed to be validated when the correlation between the excitatory synapses and inhibitory synapses is present. 

However, the specifics of a biologically plausible model of plasticity that can account for the observed synaptic patterns have remained elusive. To get biologically plausible model and remove the instability in Hebbian plasticity many mechanisms have been discussed in recent findings. One remarkable suggestion is to combine STDP with multiple dynamic and stochastic synaptic connections which enable the neurons to contact each other simultaneously through multiple synaptic communication pathways that are highly sensitive to the dynamic updates and stochastically adjust their states according to the activity history. Furthermore, strength of these individual connections between neurons is necessarily a function of the number of synaptic contacts, the probability of neurotransmitter release, and postsynaptic depolarization [20]. These synapses are further capable of adjusting their own probability of neurotransmitter release (*p*_*r*_) according to the history of short-term activity [20, 21] which provides an elegant way of introducing activity-dependent modifications to synapses and to generate the competition between synapses [22]. Based on this hypothesis many approaches have been proposed by modeling the behavior at synapses stochastically [23, 24]; here the model we have proposed differs from others because of the computational power that has been granted to the modelled receptors, so that behavior at a single synapse was determined by collective activities of these dynamic stochastic receptors. Using this model, an experiment was conducted to find the answers to the following two questions: first, can STDP and short-term plasticity control the excitation of Hebbian neurons in neural networks without weight constraints? Second, if the excitation was controlled what parameters help STDP in such a controlling activity? 

A fully connected neural network was developed with two neurons in which each neuron consisted of thousands of computational units. These computational units were categorized as transmitters and receptors according to the role they played on the network. A unit was called a transmitter if it transmitted signals to other neurons and a unit was called a receptor if it received the signals into the neuron. The receptors of a given neuron were clustered into receptor groups. According to the excitation and the inhibition of the model neuron these computational units could update their states dynamically from active state to inactive state or vice versa. Only when a computational unit was in active state it could successfully transmit signals between neurons. Transmitters from presynaptic neuron and receptors of the corresponding receptor group of the postsynaptic neuron together simulated the process of a single synapse. Transmitter at presynaptic neuron can be considered as a synaptic vesicle which can release only a single neurotransmitter at a time and the model receptors can be considered as postsynaptic receptors at synaptic cleft. With these features, excitation of a neuron at a particular synapse in our network was determined by the function of the number of active transmitters in the presynaptic neuron, transmitters' release probability, and the number of active receptors at the corresponding receptor group of the postsynaptic neuron. First, in order to analyze how network with two neurons could balance the excitation when Poisson inputs with mean rates 10 Hz and 40 Hz were applied, Only one neuron was fed by Poisson inputs while letting the other neuron to adjust itself according to the presynaptic fluctuations. Neurons stabilized its weight for both Poisson inputs while the weight values of Poisson inputs with mean rate 10 Hz were stabilized into higher range compared to when Poisson inputs with mean rate 40 Hz was applied. The analysis into internal dynamics of neurons shows that neurons have behaved similar to the process discussed in synaptic redistribution when long-term plasticity interacts with short-term depression. Further, neurons have played complementary roles to maintain the network's excitation in an operational level. These compensatory roles have not damaged the network biological plausibility as we could see that neurons worked as integrators that integrate higher synaptic weighted inputs to lower output and vice versa. Finally the network behavior was evaluated for other Poisson inputs with mean rates in the range of 10 Hz to 40 Hz and observed as the mean rate of the Poisson inputs increases, the immediate postsynaptic neuron increases its synaptic weights, while the immediate presynaptic neuron of those inputs was settle, into a complementary state to the immediate postsynaptic neuron. 

## 2. Method

**Figure 1:**
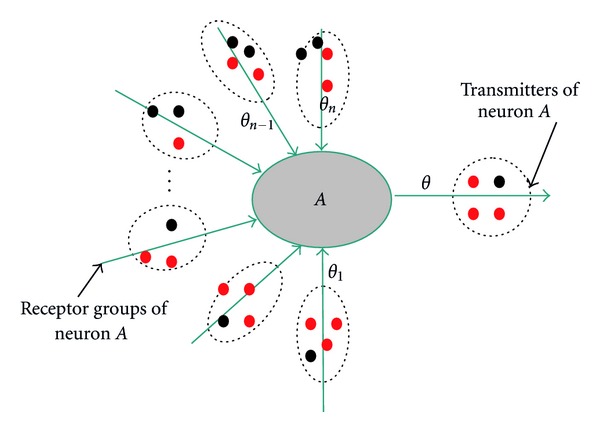
Structure of neuron *A*.

A fully connected network with two neurons was created. Each neuron was attached to thousands of computational units which were either in active state or inactive state according to the excitation and the inhibition of the attached neuron. Units attached to a neuron were classified into two groups based on the role they played on the neuron. A computational unit that transmitted the signal from the attached neurons to other neurons was called a transmitter and a computational unit that received the signals to the attached neurons from other neurons was called a receptor. Further, receptors attached to a neuron were clustered into groups so that transmitters from presynaptic neuron could contact the postsynaptic neuron simultaneously through multiple synaptic connections.  Figure 1 shows the structure of our modeled neuron *A*, with *n* receptor groups and a transmitter set. Moreover, transmitters in our presynaptic neurons were similar to the synaptic vesicles in real neurons with a single neurotransmitter. The states, either active or inactive, of these transmitters and receptors were modeled using two-state stochastic process as explained in the next section. Only when the units were in active states, they were reliable to successfully transmit or receive the signals to or from other neurons. 

**Figure 2:**
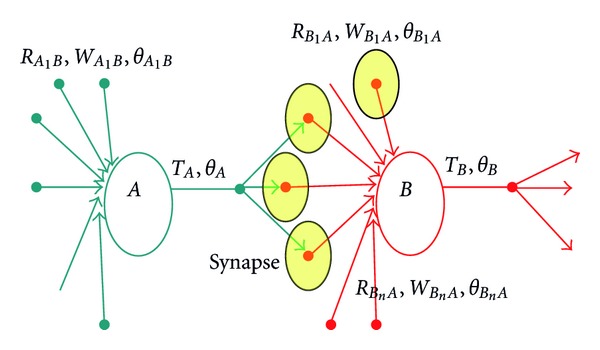
Structure of the neural network.

The transmitters from presynaptic neurons contacted the receptors of a particular receptor group of postsynaptic neurons by forming a synapse between the two neurons; see Figure 2. Through multiple receptor groups of postsynaptic neurons, presynaptic transmitters could make multiple synaptic connections simultaneously forming dynamic and stochastic synapses. As depicted in the Figure 2 each receptor group *R* of postsynaptic neuron and transmitter set *T* of presynaptic neuron jointly measured the excitation at the attached synapse *w* and balanced the excitation using threshold *θ* as discussed in the next section. 

### 2.1. Process at Dynamic Stochastic Synapses

When defining the process under dynamic stochastic synapses we have only concerned with the properties and mechanisms of use-dependent plasticity from the few milliseconds to several minutes time scales. Therefore, use-dependent activity to our modeled network was introduced using short-term plasticity; facilitation and depletion [25, 26]. When defining the probability of neurotransmitter release at our modeled transmitters it was assumed that facilitation at biological synapses depends only on the external ca^+2^ ions that influx to the biological synapse after arriving of an action potential and residual ca^+2^ ion concentrations that the synapse already has. And depletion has no influence on ca^+2^  ion concentrations and merely depends on use activity of the synapse. Then signal release probability at a transmitter in a synapse was adopted by the model proposed in [27] which determines the signal release probability *p*_*r*_ as a function of ca^+2^  ions influx to synapse, vesicle depletion, and signal arriving time to the transmitter. Only the influx of ca^+2^  ions after arriving of neurotransmitters into receptors of postsynaptic neuron was considered when determining states of the receptors. 

If *Ps*(*t*_*i*_) is the probability that signal is released by a transmitter *S* at time *t*_*i*_ and train $\underline{t}=\{t_{1},t_{2},\ldots ,t_{n},\ldots \}$ consists of exact signal releasing times of the *S*, *S*(*t*) consists of the sequences of times where *S* has successfully released the signals. The map $\underline{t}\to S(t)$ at *S* forms a stochastic process with two states, that is, Release (*R*) for *t*_*i*_ ∈ *S*(*t*) and Failure of Release (*F*) for *t*_*i*_ ∉ *S*(*t*). The probability *Ps*(*t*_*i*_) in (1) describes a signal release probability at time *t*_*i*_ by *S* as a function of facilitation *C*(*t*) ≥ 0 in (2) and a depletion *V*(*t*) > 0 in (4) at time *t*. *C*_0_ and *V*_0_ are the facilitation and depression constants, respectively. Function *C*′(*s*) given in (3) defines the response of *C*(*t*) to presynaptic signal that had reached to *S* at time *t* − *s*; *α* is the magnitude of the response. Similarly *V*′(*s*) given in (5) models the response of *V*(*t*) to the preceding releases of the synapse *S* at time *t* − *s* ≤ *t* and *τ*_*c*_ and *τ*_*v*_ are time decay constants of facilitation and depression. Maass and Zador [27] allowed *S* to release the received signal at time *t*, if *Ps*(*t*_*i*_) > 0. We updated this rule by introducing a new *θ* threshold so that if *Ps*(*t*_*i*_) > *θ*, a transmitter *S* is allowed to release the received signal. And we called it as in active state. Receptors in the postsynaptic neuron were modeled using the same model of Maass and Zador except that they were not involved in the process of vesicle depletion. Therefore, the states of the receptors were determined by setting the depletion *V*(*t*) in (1) into a unit. According to the recent biological findings of [22], parameters were initialized to *C*_0_ = 20, *V*_0_ = 10,*τ*_*c*_ = 100 ms, *τ*_*v*_ = 800 ms, and *α* = 4:

(1) Ps(ti)=1−exp⁡(−C(ti)·V(ti)),

(2) C(t)=C0+∑ti<tC′(t−ti),

(3) C′(s)=α·exp⁡(−sτc),

(4)   V(t)=max⁡(0,V0    −∑ti<t,ti∈S(t)V′(t−ti)),

(5) V′(s)=exp⁡(−sτv).

A modeled neuron maintained threshold values *θ* for each receptor groups and set of transmitters. Let *R*_*J*_*j*_*I*_ denote the *j*th receptor group in postsynaptic neuron *J* that contact transmitters in the presynaptic neuron *I*, and let *X*_*J*_*j*_*I*_(*t*) the output and *θ*_*J*_*j*_*I*_(*t*) be the threshold value of *R*_*J*_*j*_*I*_ at time step *t*. Similarly let *T*_*I*_ denote the transmitters in neuron *I*and let *O*_*I*_(*t*) be the output and *θ*_*I*_(*t*) the threshold value of *T*_*I*_ at time step *t*. The threshold value of the receptor group *R*_*J*_*j*_*I*_ was defined as in (6) and it was exponentially increased as the activity of *R*_*J*_*j*_*I*_ to *T*_*I*_ is increasing (or decreased when the activity of *R*_*J*_*j*_*I*_ to *T*_*I*_ is decreasing). Threshold value for transmitters in neuron *I*, that is, *T*_*I*_, was defined as a function of total synaptic inputs from all its synaptic connections to the neuron *I* into the total output of the neuron as in (7). Every 60 time steps threshold values of both neurons were updated:

(6) θJjI(t)=f(XJjI(t)OI(t))

Let *g* be the number of receptor groups a neuron has, then

(7) θI(t)=f(OI(t)·∑i=1gXIjJ(t))

*f*(*x*) = 1/(1 − *e* ^−*x*^), *X*_*J*_*j*_*I*_(*t*) = |*R*_*J*_*j*_*I*_^Act^(*t*)|/|*R*_*J*_*j*_*I*_|;  *i* = 1,2,…, *g*; *O*_*I*_(*t*) = |*T*_*I*_^*Act*^(*t*)|/|*T*_*I*_|.  |*G*| is the number of components in *G* and |*G* ^Act^(*t*)| is the number of active components in *G* at time step *t*. 

Moreover, according to the following predefined behavioral rule signal was propagated between neurons. 

*Rule  1*. When a receptor receives a signal from the corresponding presynaptic neuron at time step *t*, the signal is propagated within the network according to the following conditions.

**Condition 1**

Once a received signal is applied to a receptor if the receptor is updated to inactive state then the received signal is inactivated otherwise the signal is propagated to a randomly selected transmitter of the same neuron.

**Condition 2**

Once a transmitter of a particular neuron receives a signal at time step *t*, the signal is transmitted to a randomly selected receptor of the randomly selected receptor group of the postsynaptic neuron if updated state of the transmitter is active otherwise the received signal is inactivated.

The above behavioral rule defines the underlying mechanism of signal transmission between the presynaptic neuron and the postsynaptic neuron; that is, when the related computational units from the two neurons are active only, the signal is successfully transmitted. Therefore, the number of active receptors in a receptor group of the postsynaptic neuron and the number of active transmitters in the presynaptic neuron jointly define the efficacy at a given synapse. In addition to this short-term plasticity and homeostatic synaptic plasticity [28, 29] adjustments (it was shown that under similar conditions, neurons processed similar to Hebbian neurons [30] and the defined threshold mechanism functioned as a homeostatic synaptic plasticity process; see [31]) our dynamic stochastic synapses are subject to long-term plasticity induced by STDP as discussed next.

### 2.2. Bin the Process at Synapses

The process at synapses where transmitters from the presynaptic neuron contacted the receptors in a particular receptor group of the postsynaptic neuron were binned to analyze the synapse's excitation. Bin is an array of seven columns, *n*_*b*_ = 7, which stored data of a given synapse of successive seven time steps. A single cell of a bin contains data at a time step *t*, namely, the number of active transmitters in the presynaptic neuron, the number of active transmitters in the postsynaptic neuron, the number of active receptors in the corresponding receptor group of the postsynaptic neuron, and the mean release probability of transmitters in presynaptic neuron. Let *C*_*i*_ be *i*th cell of *k*th bin, the time gap between two consecutive cells is set to 5 ms as in (8). 

**Figure 3:**
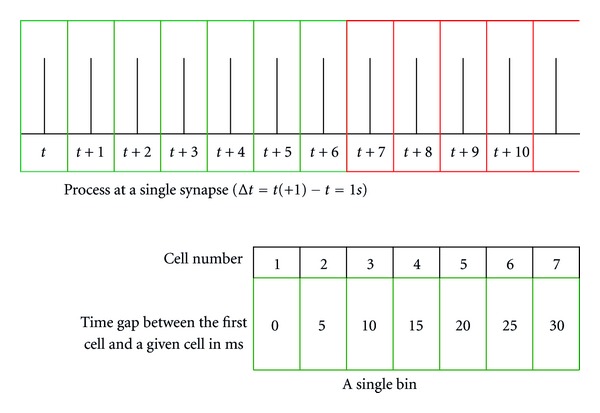
Bin the process at a single synapse.

This allowed us to define the time represented by each cell in a bin from its first cell as in (9); see Figure 3. This arrangement of bin was necessary in our model to satisfy the condition (*t*_*c*_1__ = 0 ms)<(*τ*_+_ = *τ*_−_ = 20 ms)<(*t*_*c*_7_^  ^_ = 30 ms), where *τ*_+_ and *τ*_−_ are membrane constants for potentiation and depression (discussed later):

(8) Δtci+1−ci=5  ms, i=1,…,6,

(9) tci=(0,tci)=∑i=175·(i−1).

Let $\underline{AT_{\text{pre}}}=\{AT_{\text{pre},1},AT_{\text{pre},2},\ldots ,AT_{\text{pre},n_{b}}\}$ be random variables of the number of active transmitters in presynaptic neuron at successive seven time steps of a bin; similarly let $\underline{AT_{\text{post}}}=\{AT_{\text{post},1},AT_{\text{post},2},\ldots ,AT_{\text{post},n_{b}}\}$ be random variables of the number of active transmitters in postsynaptic neuron and let $\underline{AR_{\text{post},s}}=\{AR_{\text{post},s,1},AR_{\text{post},s,1},\ldots ,AR_{\text{post},s,n_{b}}\}$ be random variables of the number of active receptors in receptor group *s* that correspons to synapse *s* in *k*th bin (*B* ^*k*^). Since the activity between the presynaptic transmitters and receptors in receptor group *s* is not independent, we defined mean, *μ*_*B*^*k*^,*s*_, and variance, *σ*_^*B*^*k*^,*s*^_^2^, of the *k*th bin on synapse *s* as in (10) and (11):

(10) μBk,s=μATpre+μARpost,s  ,

(11) σBk,s2=Var⁡(ATpre+ARpost,s)=σATpre2+σARpost,s2−2Cov(ATpre,ARpost,s),

where *μ*_*AT*_pre__ and *σ*_*AT*_pre__^2^ are the mean and variance of *AT*_pre_. Similarly, *μ*_*AR*_post,*s*__ and *σ*_*AR*_post,*s*__^2^ are the mean and variance of *AR*_post,*s*_. The mean and variance of both *AT*_pre_ and *AR*_post,*s*_ were estimated using maximum likelihood estimators, so that *μ*_*B*^*k*^,*s*_ in (10) can be written as in (12) if $\bar{AT_{\text{pre}}}=\sum_{j=1}^{n_{b}}AT_{\text{pre}}^{j}/n_{b}$ and $\bar{AR_{\text{post},s}}=\sum_{j=1}^{n_{b}}AR_{\text{post},s}^{j}/n_{b}$ are the sample means, and *σ*_*B*^*k*^,*s*_^2^ in (11) can be written as in (13) if $S_{AT_{\text{pre}}}^{2}=\sum_{j=1}^{n_{b}}\left(AT_{\text{pre}}^{j}-\bar{AT_{\text{pre}}}\right)/\left(n_{b}-1\right)$, and $S_{AR_{\text{post},s}}^{2}=\sum_{j=1}^{n_{b}}\left(AR_{\text{post},s}^{j}-\bar{AR_{\text{post},s}}\right)/\left(n_{b}-1\right)$ are the sample variances of *AT*_pre_ and *AR*_post,*s*_ respectively. The covariance of *AT*_pre_ and *AR*_post,*s*_ is defined in (14):

(12) μ^Bk,s=ATpre¯+ARpost,s¯,

(13) σBk,s2=S  ATpre2+S  ARpost,s2−2Cov(ATpre,ARpost,s),

(14) Cov(ATpre,ARpost,s)  =∑j=1nb(ATprej−ATpre¯)(ARpost,sj−ARpost,s¯)nb−1.

The mean release probability of the presynaptic transmitters within a given bin, say *B* ^*k*^, can be defined as in (15), if ${\bar{P}}_{T_{i}}$ be the mean release probability of the transmitters in presynaptic neuron at time step *t* + *i*:

(15) MB,skT=∑i=1nbP¯Tinb.

### 2.3. Defining Synapse's Activity Using Bins' Activity

STDP is a form of long-term modification to synaptic strength that depends on the action potential arriving timing between presynaptic neuron *t*_pre_ and postsynaptic neuron *t*_post_ [4] and can be described by weight window function defined in (16). This weight function defines how strength between the two neurons can be adjusted for a single pair of action potential within the time window Δ*t* = |*t*_pre_ − *t*_post_|. As defined in (16) if presynaptic action potential occurs before the postsynaptic action potential then it strengths the synaptic strength and called long-term potentiation. Conversely if postsynaptic potential occurs before the postsynaptic action potential, it weakens the synaptic strength and called long-term depression:

(16) W(Δt)={A+·e−(tpost−tpre)/τ+if  tpre<tpost,−A−·e−(tpre−tpost)/τ−if  tpre≥tpost.

Here *A*_+_, *A*_−_ > 0 and *τ*_+_, *τ*___ are membrane constants of long-term potentiation and long-term depression. The values for *A*_+_ and *A*_−_ need to satisfy the condition *A*_+_ *τ*_+_ < *A*_−_ *τ*_−_ as it required the integral of the weight window to be negative to generate stable synaptic strength based on STDP [4]. Furthermore, recent biologically observations [32] have estimated *τ*_+_ and *τ*_−_ roughly to 20 ms. Thus, in order to generate stable synaptic strength, it is required to have *A*_+_ < *A*_−_. In our model, the weight window function was applied in bin level at each synapse in order to apply long-term modifications to neuron. Let *H*__pre,*c*_pre___^*B*_*k*_^ be the highest amount of active transmitters recorded from the presynaptic neuron during bin *B* ^*k*^ and it was at cell *c*_pre_ as defined in (17). Similarly let *H*__post,*c*_post___^*B*_*k*_^ be the highest amount of active transmitters recorded from the postsynaptic neuron during bin *B* ^*k*^ and it was at cell *c*_post_ as defined in (18). Then STDP weight window function was applied on bin's level by mapping *H*__pre,*c*_pre___^*B*_*k*_^ as an action potential occurred in the presynaptic neuron during bin *B* ^*k*^ which could significantly update the synaptic strength presynaptically at the corresponding synapse and *H*__post,*c*_post___^*B*_*k*_^ as the action potential occurred in the postsynaptic neuron during bin *B* ^*k*^ which could significantly update the synaptic strength postsynaptically on the same synapse. Here we have assumed that within the duration of a bin only the highest hitter of that bin can significantly update the synaptic strength. Subsequently we mapped *t*_pre_ to (*c*_pre_ − 1) × 5  ms and *t*_post_ to (*c*_post_ − 1) × 5  ms. Therefore, if postsynaptic hitter occurs after the presynaptic hitter, it leads to the potentiation, and if presynaptic hitter is followed by the postsynaptic hitter, it depresses the synapses during the given bin:

(17) ∀i  ATpre,i>ATpre.j  , i=1,…,7,  j=1,…,7,  i≠j

(18) ∀i  ATpost,i>ATpost,j, i=1…7,  j=1…7,  i≠j

### 2.4. Mean and Variance of a Synapse

Learning based on STDP was implemented on synapses assuming that bins of a given synapse are mutually independent and the impact that each bin made on the synapse sums linearly. Then mean *μ*_*S*_*k*_^*s*^_ and variance *σ*_*S*_*k*_^*s*^_^2^ of the *s*th synapse (*S* ^*s*^) can be defined as in (19) and (20) when *k*th bin (*B* ^*k*^) is interacted with the synapse. The mean *μ*_*S*_*k*_^*s*^_ and the variance *σ*_*S*_*k*_^*s*^_^2^ of the synapse *S* ^*s*^ were estimated using maximum likelihood estimators as shown in (21) and (22), respectively. Further, the total mean release probability at synapse *S* ^*s*^ at *B* ^*k*^ was defined using bin's mean release probabilities as in (23):

(19) μSks=μSk−1s+μBk,s; μS0s=0,    k=1,2,…,    

(20) σSks2=Var⁡(Sk−1s+Bk)=σSk−1s2+σBk,s2;σS0s2=0, k=1,2,…,

(21) μ^Sks=μ^Sk−1s+μ^Bk,s

(22) σ^Sks2=σ^Sk−1s2+σ^Bk,s2

(23) MSksT=MSk−1sT+MBk,s; MS0sT=0,  k=1,2,….

  In order to generate action potentials real neurons are necessary to be in a nonquiescence state. If a neuron is in a quiescence state, it is not possible for the neuron to generate action potentials that can change the synaptic strength significantly. Therefore, STDP was applied on synapses only if model presynaptic and postsynaptic neurons were not in quiescence states. We defined that a neuron is not in a quiescence state when the average output produced by the neuron during bin *B* ^*k*^ is greater than the average output it had produced so far. That is, if the current mean number of active transmitters of a particular neuron is less than the mean number of active transmitters during the *k*th bin, neuron was recognized as in a nonquiescence state at bin *B* ^*k*^. That is mathematically if *M*_*AT*_pre__^*S*_*k*−1_^*s*^^ ≤ *M*_*AT*_pre_^*B*^*k*^^_ and *M*_*AT*_post__^*S*_*k*−1_^*s*^^ ≤ *M*_*AT*_post_^*B*^*k*^^_, the weight was updated on the synapse *S* ^*s*^ at bin *B* ^*k*^ as discussed next.

### 2.5. Learning Based on STDP and Release Probability

According to the model proposed in [33], the amplitude of the excitatory postsynaptic current *A*_*k*_ of the *k*th spike in a spike train is proportional to the weight at that synapse and the release probability at the *k*th spike. In our approach *A*_*k*_ is proportional to the impact that made by transmitters in the presynaptic neuron and receptors in the corresponding receptor group of the postsynaptic neuron during *B* ^*k*^ on the synapse *S* ^*s*^. If we applied the model proposed in [33] to our *k*th bin instead of *k*th spike, we can express *A*_*k*_ as in (24). Moreover, biological evidence supports the fact that the amount of change on weight is also dependent on the initial synaptic size [34]. Depression is independent of the synaptic strength, whereas strong synapses are less potentiated than weak synapses. By assuming that there is an inverse relationship between the initial synaptic strength and the amount of potentiation, potentiation can be expressed for the *k*th bin, (*B* ^*k*^) as in (25) if *W*_*S*^*s*^,*k*_^*p*^ is the amount of potentiation during *k*th bin at synapse *S* ^*s*^:

(24) Ak,Ss∝Wk,Ss·Uk,Ss,

(25) Wk,Ss∝1Wk,Ssp.

If we combined (16), (24), and (25), the amount of weight updated during *k*th bin at synapse *S* ^*s*^, *W*_*S*^*s*^_(Δ*k*), can be defined as in (26) and the synaptic weight at *S* ^*s*^ at the end of bin *B* ^*k*^ is determined as in (27):

(26) WSs(Δk)={γp·Ak,SsUk,Ss·1Wk,Ssp·e−(tpost−t  pre  )/τ+if  tpre<tpost−γd·Ak,SsUk,Ss·e−(tpre−t  post  )/τ−if  tpre≥tpost    k=1…,  s=1…

(27) WSs(k)=WSs(k−1)+WSs(Δk),

where *γ*_*p*_ = 0.005 and *γ*_*d*_ = 0.00525 are potentiation and depression learning rates [11]. The amplitude *A*_*k*_ during the *k*th bin was estimated by the proportion of the deviation that made by the bin compared to its mean, to the deviation that synapse had made so far compared to its overall mean. That is, in statistically amplitude *A*_*k*_ during the *k*th bin can be expressed as a proportion of the coefficient variation (CV = *σ*/*μ*) during the *k*th bin to the coefficient variation of the synapse *S* ^*s*^ has as given in (28). The release probability *U*_*k*,*S*^*s*^_ during the *k*th bin was determined as a proportion of mean release probability during *k*th bin to the total mean release probability at synapse *S* ^*s*^ has as in (29). Median of the weight distribution at synapse *S* ^*s*^ was taken as an estimator for *W*_*k*,*S*^*s*^_^*p*^ as in (30). This is merely because median provides a good approximation about the center of the weight distribution than mean; that is, the median is not affected by the outliers, whereas the mean is affected by the outliers:

(28) Ak,SsS=CVBk,sCVSs=(σBk,s2/μBk,s)(σSks2/μSks); k=1…,  s=1…    

(29) Uk,Ss=MBk,sTMSksT; k=1,…,  s=1…

(30) Wk,Ssp=median{WSs(i) ∣ i=1,…,k−1,  s=1…}.

## 3. Balancing the Excitation of the Network

**Figure 4:**
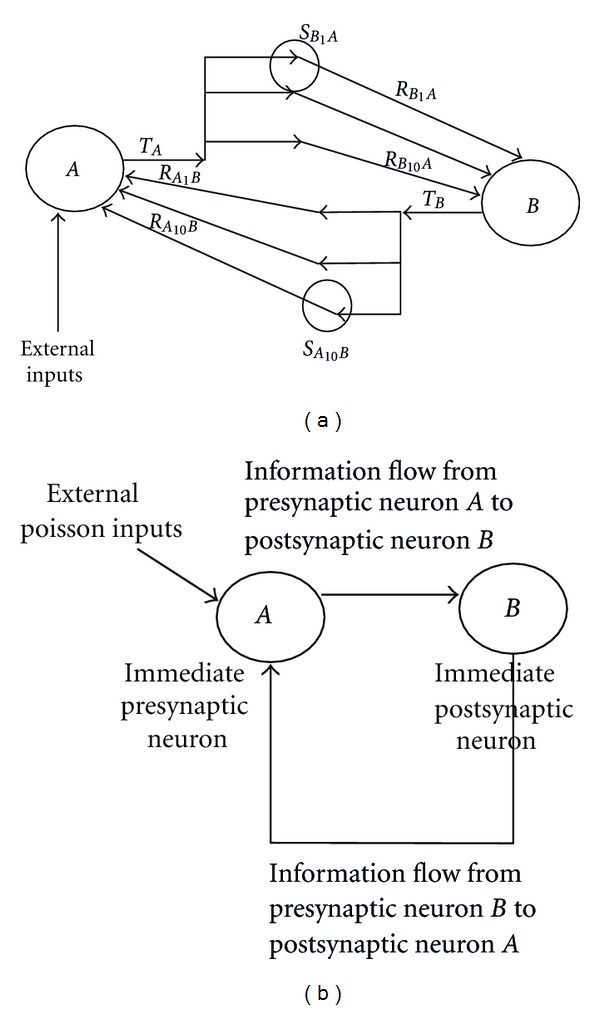
Network structure with ten synaptic connections. (a) shows the developed fully connected network to test how neurons could balance the excitation after external input was applied to part of it. *T*_*A*_ and *T*_*B*_ are the outputs (the number of active transmitters attached to the neuron at a given time step) of neuron *A* and neuron *B*, respectively. Receptor groups *R*_*AB*_ and receptor groups *R*_*BA*_ symbolized the number of active receptors in the corresponding receptor groups of postsynaptic neuron *A* and postsynaptic neuron *B*, respectively. *S*_*AB*_ are the synapses where receptor groups of postsynaptic neuron *A *contact the transmitters of neuron *B. *Similarly, *S*_*BA*_ are the synapses where receptor groups of postsynaptic neuron *B* contact the transmitters of neuron *A.* (b) shows how information flows between the two neurons. When signals are passing from *A* to *B, A* is called presynaptic neuron and *B *is called postsynaptic neuron. Similarly, when signals are passing from *B* to *A, B *is called presynaptic neuron and *A* is called postsynaptic neuron. Since external Poisson inputs are applied to neuron *A* only, neuron *A* becomes the immediate presynaptic neuron and *B *becomes the immediate postsynaptic neuron for the external inputs.

An experiment was arranged to find the possibility that can STDP and short-term plasticity together balance the excitation of a network with two Hebbian neurons without defining any constraints on the weight learning algorithm. A fully connected network with two neurons, say neuron *A* and neuron *B*, was developed; each neuron had ten receptor groups, making a presynaptic neuron to contact the postsynaptic neuron through ten dynamic stochastic synapses simultaneously. Both neurons, *A* and *B* had equal number of transmitters *n*_*T*_ = 30000 and receptors *n*_*R*_ = 30000; and receptors attached to neurons were uniformly distributed among receptor groups. At the onset one percent of transmitters and one percent of receptors in each receptor-group was set to active state. Poisson inputs with mean firing rates (*λ*) 10 Hz and 40 Hz were applied to all the receptor groups of neuron *A*simultaneously while giving enough space for neuron *B* to adjust itself according to the feedbacks of neuron *A*,see Figure 4. Each input was applied around two hours continuously to neuron *A*; and the behavior of the network was analyzed after the synaptic connections have established the effect of the altered activity and network activity has developed. The values were fed to the system according to following rule: the generated Poisson distribution was converted to byte stream by the following rule: if generated value is greater than the median of the Poisson distribution then it was considered to represent value 1 otherwise it was considered to represent value 0. Only when the represented value is equal to one, the signal was generated and fed to neuron *A*.

**Figure 5:**
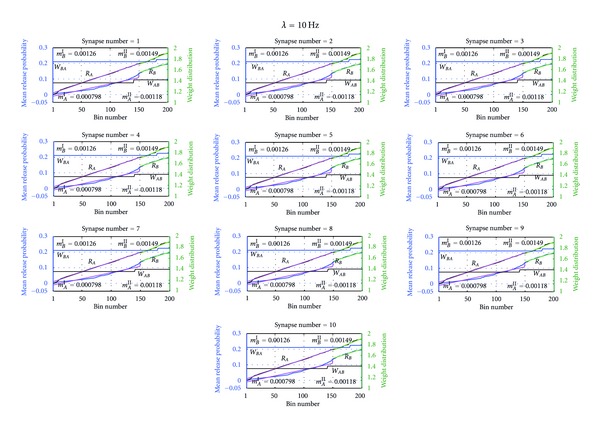
Distribution of the weights and release probabilities of neurons at Poisson inputs with mean rate 10 Hz. Each subfigure in the figure depicts the distribution of the weight algorithm and the mean release probability at the given synapse of postsynaptic neuron *A *and postsynaptic neuron *B* at Poisson inputs with mean firing rate 10 Hz.For example, the leftmost top subfigure shows the variation of the mean release probability and the weight distribution at the first synapse of the ten synapses. As shown in the figure, the network, both neuron *A* and neuron *B,* spent around 150 bins to adjust to the external Poisson inputs and subsequently reach the stability. *W*_*BA*_ gives the distribution of the weights of the synaptic connections from presynaptic neuron *A *to postsynaptic neuron *B*. Similarly *W*_*AB*_ gives the distribution of the weights of the synaptic connections from presynaptic neuron *B* to postsynaptic neuron *A*. *R*_*B*_ is the distribution of the mean release probability of transmitters of presynaptic neuron *B *and *R*_*A*_ is the distribution of mean release probability of transmitters of presynaptic neuron *A* at postsynaptic connections of neuron *B*. Moreover, the slopes of the mean release probabilities, *m*_*B*_^*I*^ and *m*_*B*_^*II*^, were determined using linear regression analysis and give the slope of mean release probability of *S*_*BA*_ from bin 1 to 150 and bin 150 to 200, respectively. Similarly, *m*_*A*_^*I*^ and *m*_*A*_^*II*^ give the mean release probability of *S*_*AB*_ from bin 1 to bin 150 and from 150 to 200, respectively.

**Figure 6:**
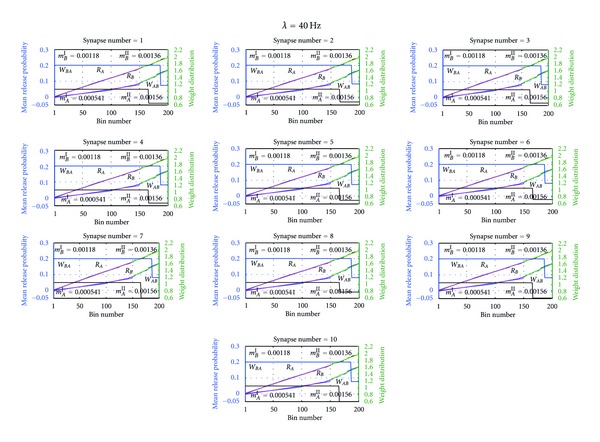
Distribution of the weights and release probabilities of neurons at Poisson inputs with mean rate 40 Hz. Each subfigure in the figure depicts the distribution of the weight algorithm and the mean release probability at the given synapse of postsynaptic neuron *A *and postsynaptic neuron *B* at Poisson inputs with mean firing rate 40 Hz. For example, the leftmost top subfigure shows the variation of the mean release probability and the weight distribution at the first synapse of the ten synapses. As shown in the figure, the network, both neuron *A* and neuron *B*, spent around 150 bins to adjust to the external Poisson inputs and subsequently reach the stability. *W*_*BA*_ gives the distribution of the weights of the synaptic connections from presynaptic neuron *A *to postsynaptic neuron *B*. Similarly, *W*_*AB*_ gives the distribution of the weights of the synaptic connections from presynaptic neuron *B* to postsynaptic neuron *A*. *R*_*B*_ is the distribution of the mean release probability of transmitters of presynaptic neuron *B *and *R*_*A*_ is the distribution of mean release probability of transmitters of presynaptic neuron *A* at postsynaptic connections of neuron *B.* Moreover, the slopes of the mean release probabilities, *m*_*B*_^*I*^ and *m*_*B*_^*II*^, were determined using linear regression analysis and give the slope of mean release probability of *S*_*BA*_from bin 1 to 150 and bin 150 to 200, respectively; Similarly, *m*_*A*_^*I*^ and *m*_*A*_^*II*^ give the mean release probability of *S*_*AB*_ from bin 1 to bin 150 and from 150 to 200, respectively.

**Figure 7:**
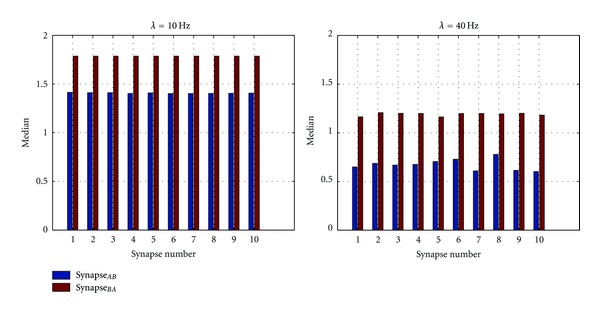
Distribution of the medians of synaptic weights at each synapse at Poisson inputs. The figure shows the variation of the median of the weight distribution at each Poisson input; *λ* is the mean Poisson firing rate. The median in the above figures was determined after synaptic connections have established the effect of external inputs and network stabilized. This stabilization happened after 170 bins; see Figure 5. Medians of postsynaptic neuron *B*, Synapse_*BA*_ show the variations of the medians of the weight distributions of synaptic connection from presynaptic neuron *A *to postsynaptic neuron *B.* Similarly, Synapse_*AB*_ show the variations of the median of the weight distributions of synaptic connections from presynaptic neuron *B *to the postsynaptic neuron *A*.

Figures 5 and 6 show the distribution of weights of both postsynaptic neurons *A* and *B *at Poisson inputs with mean rates 10 Hz and 40 Hz. As shown in these figures, the weight distributions of both neurons at each, synapse have stabilized around 175 bins. After the weights distribution was stabilized, the median of the weight distribution was calculated and these calculated median values are shown in Figure 7. As shown in the figure of Poisson inputs with low mean rate, that is, at 10 Hz, medians of the all the synapses of the postsynaptic neurons reach to higher value compared to when Poisson inputs with higher mean rate, that is, at 40 Hz, were applied. The network balanced its excitation by pushing synaptic weights towards higher values for inputs with low mean rate and for inputs with higher mean rate the network has pulled down the synaptic weights into a lower weight values. This dynamic behavior of both neurons is a necessary adjustment to balance neurons' excitation and subsequently to balance the network excitation while adjusting to external manipulations. 

**Figure 8:**
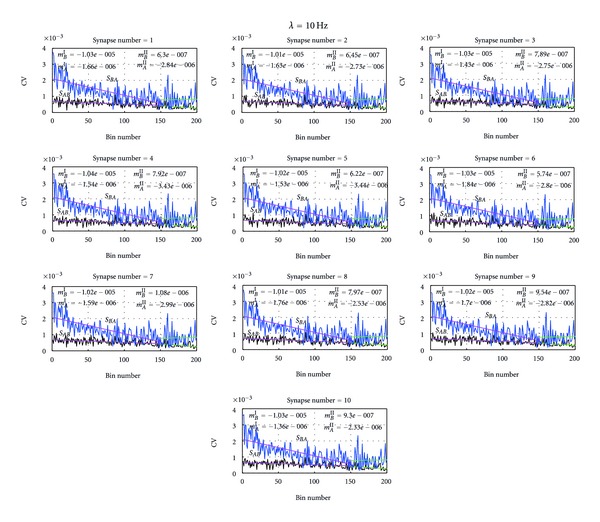
Distribution of the coefficient of variation (CV) at each synapse at Poisson inputs with 10 Hz. Each subfigure Figure 8 depicts the distribution of the CV at the given synapse of both neuron *A *and neuron *B* at Poisson inputs mean firing rate 10 Hz. For example, As shown in the leftmost subfigure *S*_*AB*_ shows the variation of CV of synapses from presynaptic neuron *B* to postsynaptic neuron *A.* Similarly, *S*_*BA*_ depicts the distribution of CV of synapses from presynaptic neuron *A* to postsynaptic neuron *B.* Moreover, the slopes of the mean release probability, *m*_*B*_^*I*^ and *m*_*B*_^*II*^, were determined using linear regression analysis and give the slope of CV of *S*_*BA*_from bin 1 to 150 and bin 150 to 200, respectively. Similarly, *m*_*A*_^*I*^ and *m*_*A*_^*II*^ give the CV of *S*_*AB*_ from bin 1 to bin 150 and from 150 to 200, respectively. As depicted in all these subfigures, at all the synapses when its around 200 bins, the difference of the CVs of neuron *A* and neuron *B* was about 0.001 and time functions of CVs of both neurons were paralleled to each other.

**Figure 9:**
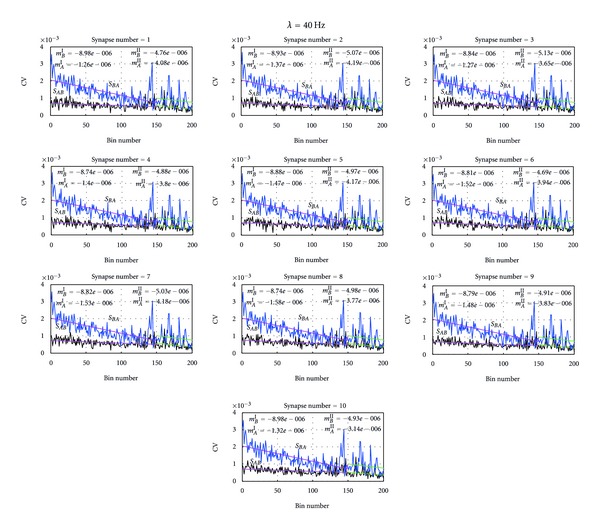
Distribution of the CV at each synapse at Poisson inputs with mean firing rate 40 Hz. Each subfigure in the figure depicts the distribution of the CV at the given synapse of both neuron *A *and neuron *B* at Poisson inputs mean firing rate 40 Hz. For example, As shown in the leftmost subfigure *S*_*AB*_ shows the variation of CV of synapses from presynaptic neuron *B* to postsynaptic neuron *A.* Similarly, *S*_*BA*_ depicts the distribution of CV of synapses from presynaptic neuron *A* to postsynaptic neuron *B.* Moreover, the slopes of the mean release probability, *m*_*B*_^*I*^ and *m*_*B*_^*II*^, were determined using linear regression analysis and give the slope of CV of *S*_*BA*_ from bin 1 to 150 and bin 150 to 200, respectively. Similarly, *m*_*A*_^*I*^ and *m*_*A*_^*II*^ give the CV of *S*_*AB*_from bin 1 to bin 150 and from 150 to 200, respectively. As depicted in all these subfigures, at all the synapses when its around 200 bins, the difference of the CVs of neuron *A* and neuron *B* was about 0.001 and time functions of CVs of both neurons were paralleled to each other.

Next we were interested to know what makes the neuron to stabilize its activity without being overexcited or overdepressed in a network which has no controlling constraints. To understand that we analyzed the internal behaviors of neurons *A* and *B* in terms of their mean release probability (the mean of the release probabilities of transmitters attached to the neuron) and the coefficient variation which measures the given synapse's excitation (CV = CV_*B*^*k*^,*s*_ in (28)) in terms of the number of active transmitters in the presynaptic neuron and the number of active receptors in the corresponding receptor groups of the synapse. Thus, the value of CV shows the extent of variability of the given synapse in relation to the synapse's mean and portraits effectively the synapse's internal dynamics. So that higher CV value implies the higher internal fluctuations and higher deviation of the synaptic mean.  Figure 5 shows the mean release probability of the presynaptic neurons at 10 Hz while Figure 8 depicts what is happening inside the synapse in terms of CV at 10 Hz. As shown in these figures, the neuron that has made higher synaptic weight has maintained higher CV compared to the other neuron in the network. Notably, the neuron which had the higher synaptic weight has produced a lower mean release probability. For example, if we analyze the behavior of neuron *B*, as shown in Figure 7, its synapses *S*_*BA*_, from presynaptic neuron *A* to postsynaptic neuron *B*, have scored higher synaptic weights at 10 Hz compared to synaptic weights of postsynaptic neuron *A*. Further, neuron *B*has maintained higher CV at all its synapses *S*_*BA*_ compared to the values of CV of the synapses of postsynaptic neuron *A*, that is, *S*_*AB*_. In contrast, the neuron *B* has maintained lower mean release probability as a presynaptic neuron at 10 Hz of Poisson inputs at its all synapses compared to neuron *A*. These opposing and balancing behaviors of the two neurons are consistent in Poisson inputs with mean firing rate 40 Hz as given in Figures 6 and 9. 

Moreover, if the difference of the value of CVs between two neurons was considered, it is clearly shown in the Figures 8 and 9 that this difference was reduced to amount 0.0001 after the two neurons have adjusted to the external input and stabilized themselves. However, most importantly, even that we could see the stabilize activity of the two neurons in terms of synaptic weights and CV the mean release probabilities have not reached to any stable position, but instead either continuously positively or negatively increasing. The positive correlation between the synaptic weights and the CV, and the negative correlation between the synaptic weights and the mean release probability at a same neuron have proven that neurons could act as integrators that integrate the excited synaptic weights and controlled the excitation via higher CV fluctuations and produced balanced output that help to balance the network activity. This reduced excitation in the output flow that has allowed the other neuron to play compensatory role and to balance the network activity. 

**Figure 10:**
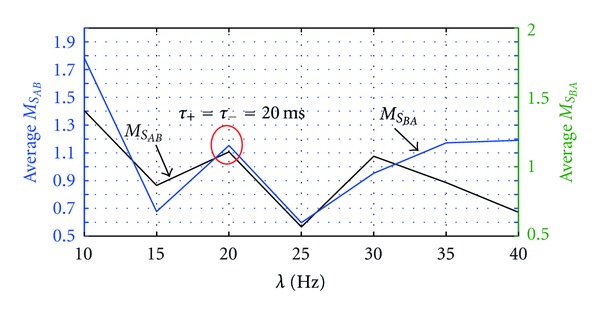
Distribution of the median of synaptic weights of neurons at different mean firing rates.  Figure 10 illustrates the average of the weight medians calculated after both neurons were stabilized after applying Poisson inputs with mean rate *λ*.  *M*_*S*_*AB*__ is the average of the medians value of all the synapses of postsynaptic neuron *A* from presynaptic neuron *B*, and similarly *M*_*S*_*BA*__is the average of the medians value of all the synapses of postsynaptic neuron *B *to presynaptic neuron *A*. *τ*_+_ = *τ*_−_ = 20 ms are STDP time constants for potentiation and depression.

Finally we would like to understand the behavior of the network when applying Poisson inputs in the range of 10 Hz and 40 Hz. The Poisson inputs with mean rates, 15 Hz, 20 Hz, 25 Hz, 30 Hz, and 35 Hz were also presented to neuron *A*'s receptor groups and studied the behavior of the both neurons on the same network.  Figure 10 shows the average value of the medians of synapses of each neuron. When magnitude of the Poisson inputs mean rate is greater than the STDP potentiation and depression time constants 25 < *λ*, the medians of the stabilized synaptic weights of synapses of neuron *B* as the immediate postsynaptic neurons of external inputs have continuously increased as the mean rate of Poisson inputs is increased. Again the compensatory behavior from neuron *A* could be seen as it has generally decreased its medians of the stabilized synaptic weights as the mean rate is increasing. These complementary behaviors of two neurons seem to be necessary to stabilize the overall network activity. When 20 > *λ*, both neurons have worked together to control the overall excitement of the network. Intriguingly, when  *λ* = 20 and *λ* = 25, the excitation of the entire network was equally balanced between the two neurons as their average value of the medians of the stabilized synaptic weights becomes almost equal to each other. This might be because of the effect of *τ*_+_ = *τ*___ = 20 ms that we selected for STDP potentiation and depression time constant. This is important observation which provides the possibilitythat postsynaptic neurons could excited and stabilized into the same level of the presynaptic neuron if STDP time constants are highly correlated with the mean rate of the Poisson inputs applied. Therefore, STDP with different time constants for potentiation and depression might be a good solution to scale down external inputs effectively into neuronal level. 

## 4. Discussion

As per the literature, a synapse can be strengthened either by increasing the probability of transmitter release presynaptically or by increasing the number of active receptors postsynaptically. This general functionality at the synapses can be varied by the interplay between long-term plasticity and short-term dynamics, especially short-term depression. Short-term depression is mainly based on vesicle depletion which is the use-dependent reduction of neurotransmitter release in the readily releasable pool [4]. When long-term plasticity is interacting with the short-term depression it is called synaptic redistribution [7]. The role of this synaptic redistribution is not yet clearly identified. However, this synaptic redistribution allows the presynaptic neuron to increase the probability of release and thereby increase the signal transmission between the two neurons. In our developed network, the two neurons have simulated a behavior similar to the effect of synaptic redistribution. Neuron *B*as the immediate postsynaptic neuron of the external inputs has scored the higher synaptic weights compared to neuron *A*. That is, its synapses, where the transmitters from immediate presynaptic neuron *A* contacted each receptor group of the postsynaptic neuron *B*, have scored higher weights compared to the other neuron. The presynaptic neuron *A* in this functional process has maintained higher mean release probability. Therefore, first, the synaptic weights of neuron *B*have been increased presynaptically by increasing the probability of neurotransmitter release. Second, the analysis of CV of these synapses of postsynaptic neuron *B* shows that it is laying higher range than the function of CV of neuron *A*, confirming the possibility of increasing the synaptic weights by higher turnover rate of active receptor component of postsynaptic neuron. This behavior of postsynaptic neuron *B* is also supported at Poisson inputs with mean firing rate 40 Hz. Intriguingly, if the behavior of postsynaptic neuron *B* at 40 Hz after neuron has adjusted to the external inputs and stabilized, was analyzed, the higher fluctuations of CV and comparatively lesser synaptic weights of neuron *B* at Poisson inputs with mean rate 40 Hz were observed compared to the postsynaptic neuron *B*'s behavior at Poisson inputs with mean rate 10 Hz, showing the possibility that synaptic redistribution can increase the synaptic weights for Poisson inputs with low mean rate at steady state and not for Poisson inputs for higher mean rate. And for higher mean rates it is only a higher turnover rate of active receptor components. Further, STDP potentiation and depression time constants have made higher impact on the behavior of those two neurons; that is, it has controlled the level of excitation of each neuron equally when the magnitude of the mean rate laid near the magnitude of the STDP time constants. As the mean rate of the Poisson inputs is increasing, the complementary roles have been initiated into the two neurons.

STDP has successfully interplayed with short-term plasticity to control the excitation or inhibition of neural network according to external adjustments. Notably, these adjustments are consistent and are also biologically plausible. The stabilization of synaptic weights in an operational level without controlling constraints seems to be possible if STDP as long-term plasticity interacts with the short-term dynamics. The dynamic behavior of short-term activity is necessary to propagate and balance the excitation of neural network without damaging the synaptic weight distribution; similar to how CV and probability of release have played with STDP to balance the excitation. When compared to Luz and Shamir [19] findings, instead of specifically using inhibitory neurons to generate negative feedback to stabilize the excitation and inhibition of the network, here we have used ground plasticity mechanisms that observed in biology to alleviate the excitation and inhibition of the network. Even though two approaches have used different derivatives of temporally asymmetric STDP to implement the stochastic response of neurons, both have once again proven the possibility of stabilization of the network excitation due to Hebbian plasticity using STDP. However, our approach differs from their mechanism because of the integration of the sensitivity of the release probability and turnover rate of active components attached to a synapse. Instead of the inhibition made on network by the inhibitory neuron by negative feedback to impinge the excitation generate by excited correlated spikes, our mechanism absorbed the high firing frequency excitation or overcomes the low firing frequency inhibition in terms of appropriate turnover rate of active components attached to a given synapse and adjusting release probabilities of the attached active transmitters. The mechanism under this manipulation of excitation and inhibition is similar to the synaptic redistribution discussed in biology, which drives our approach more towards to the biological plausibility. However, both systems are still needed to be evaluated and examined on larger networks and the approach of Luz and Shamir [19] needs to be tested when correlation between the inhibitory and excitatory synapses is present. Moreover, we compare the result of [11] against our findings on how excitation was balanced. In their model excitation was balanced by introducing synaptic competition in which synapses competed against each other to control the postsynaptic firing times; further, this competition was introduced by scaling the synaptic efficacy using hard boundary conditions. This model balanced the excitation of Poisson inputs 10 Hz and 40 Hz, so that for 10 Hz more synapses approach to the higher limit of synaptic efficacy and for higher inputs more synapses remained in lower limits. However, once the system reached stability it was hardly disturbed by the presynaptic firing frequency. Therefore, the stability that the system reached is moderately stronger than in our case. Although our model also exhibited similar characteristics at Poisson inputs 10 Hz and 40 Hz, no boundary conditions were defined to achieve this stability. Furthermore, compared to their moderately strong equilibrium discussed in terms of synaptic efficacy, internal dynamics of our neurons were continuously fluctuating around the equilibrium allowing neurons to remain dynamically active even at the equilibrium as similar to many natural systems. 

The model proposed in this research is a computational model to investigate the internal dynamics of neural networks when STDP, Hebbian plasticity and short-term plasticity, are interacting with each other. The model itself has few drawbacks; mainly the neural network of our model has spent around 150 bins to show the adjustment to external modifications; this is mainly because we selected the median of the weight distribution as the amount of synaptic potentiated as a response to the pair of presynaptic and postsynaptic spike (in (30)). This static quantifier is less sensitive to the sudden changes that occurred in the tail of the distribution until those changes are visible via many elements of the distribution. On the other hand, this quantifier effectively quantifies the distribution of the network into a range where many elements of the distribution are approximately lying. Even though mean could also be a good option for such an indicator, is very sensitive to the sudden changes and could easily forget the history of the distribution. Therefore median is better than the mean, but still necessary to look for an unbiased statistical quantifier to estimate the amount of potentiated as a response to presynaptic and postsynaptic spike pair which can represent the history as well as the sudden changes to the weight distribution effectively. The other main drawback we see in our approach is the use of bins to chunk the process at synapses. The size of the bin might be a possible constraint that also limits the performances of STDP on short-term dynamics. However, the model proposed in this research successfully balanced the synaptic excitation of the two neurons in an operational level without damaging their biological plausibility.
